# Successes and challenges of using a peer Mentor model for nutrition education within a food pantry: a qualitative study

**DOI:** 10.1186/s40795-020-00352-9

**Published:** 2020-07-14

**Authors:** Tracy L. Oliver, Amy McKeever, Rebecca Shenkman, Lisa K. Diewald

**Affiliations:** MacDonald Center for Obesity Prevention and Education, Villanova University M. Louise Fitzpatrick College of Nursing, 800 Lancaster Avenue, Villanova, PA 19085 USA

**Keywords:** Peer mentors, Nutrition education, Food insecurity, Emergency food pantry

## Abstract

**Background:**

Delivering nutrition education within an emergency food pantry (EFP) provides an opportunity to reach many food insecure households and underserved populations. However, little is known about using a peer mentor model, “*Community Cooks,”* as a modality to deliver nutrition education within this setting. This research aimed to identify the successes and challenges of using a peer mentor model within an EFP to better understand the best approaches to deliver nutrition education among community residents.

**Methods:**

In spring 2018, semi-structured interviews were conducted with 11 peer mentors after they delivered a series of nutrition workshops to community members of the EFP. Interviews were audio-recorded and transcribed verbatim. Qualitative content analysis was used to analyze the data.

**Results:**

All peer mentors were women over 40 years-of-age, were recruited from the EFP community; most were high school graduates and currently received some form of federal nutrition assistance. All peer mentors reported that the *“Community Cooks”* nutrition education program offered many benefits. Key successes of the program included serving in the role as a peer mentor was an empowering experience which gave them a sense of community, purpose, and camaraderie; 2) the nutrition education was appropriately tailored towards those living with food insecurity; 3) the recipes required minimal cooking skills and included low-cost easily accessible foods available at the EFP. Key challenges of the program were the lack of community member engagement in the nutrition education workshops.

**Conclusion:**

Challenges continue to exist when delivering nutrition education within a community EFP setting. While the use of peer mentors to deliver nutrition education messages is promising, more research is needed to quantify the impact of using a peer mentor model in underserved and food insecure communities.

## Background

Food insecurity is defined as reduced access to a sufficient quantity and quality of food, which limits the variety or desirability of one’s diet [1]. Access to healthy food is a fundamental human right, yet food insecurity remains a significant public health problem across the United States, with an estimated 37 million individuals experiencing some level of food insecurity [1]. Hunger, poverty, and food insecurity consequently influence individual health and well-being [2, 3]. Well documented in the literature are the relationships between socioeconomic status, race, and food insecurity [3]. The consequences of food insecurity include lower economic productivity, increased co-morbidities in adults, and increased learning/developmental concerns in children [3]. Rates of food insecurity for men, women, and children continue to impact many families, with an estimated 9.7 million adults and 6.5 million children living in food-insecure households [1]. These families experience unreliable income and unexpected financial burdens and may qualify for federal food assistance programs; however, they may also rely on food banks and emergency food pantries to help ends meet during difficult times [4].

According to the 2014 Hunger in America Report, it was determined that over 84% of US households who utilized EFPs were food insecure [4]. The concept of an emergency food pantries (EFPs) was developed in the late 1960s in the United States and originated in a church using the support of a faith-based model to provide food and social support [5]. The purpose of the “emergency” food pantry was to serve families on a short-term basis, however, due to more significant economic and social issues, this trend has changed, and many pantries now provide services long-term [5]. Pantries offer an invaluable source of supplemental food, at no cost, to fill the gap when other costs of living limit funds available for the purchase of food [6]. Communities who are reliant on supplemental food resources are often limited in food choices, notably healthy choices [7]. As a result, communities that rely on supplemental food have been shown to have poor diet quality along with increased rates of chronic disease, including obesity, metabolic syndrome, cardiovascular disease, hypertension, and diabetes [1, 8–12].

Since the inception of the food pantry model 60 years ago, EFPs have attempted to expand their services to address the social networks and environmental triggers that influence food insecurity [5]. These services target how to move families towards food security and self-sufficiency while continuing to provide food. As a result, some EFPs may offer a comprehensive model that includes nutrition education [13]. Other EFPs have implemented programming that addresses the diverse needs of their communities, including specific and cultural food preferences, general health and wellness care, and assistance in household and food budgeting skills [14, 15]. While there have been previous innovative nutrition education programs in the literature, gaps exist as to the best method of education delivery, and by whom, to effectively impact an EFP patron’s food knowledge and healthy eating behaviors.

Peer mentors, community health promoters, and health educators are viewed as leaders in their communities and have been used successfully worldwide to educate and promote health [16, 17]. The peer mentoring recruitment process intentionally seeks out mentors who are of a similar age or have had similar life experiences as their target audience. Peer mentors who are also members of the same community often share similar cultural beliefs, practices, and experiential knowledge that deepen a shared understanding of the challenges faced by community members [16–18]. The peer mentor and mentee connection also foster relationships that an outside expert could never accomplish. Due to this unique relationship, peer mentors can be effective in many settings from academic to low-income community settings, where health education is particularly pertinent and where behavioral interventions can be measured [17, 19]. Peer mentors have also been found to be successful when appropriately trained in nutrition to deliver health information targeting children or in facilitating changes in health-related behaviors, including physical activity, smoking, and condom use in both adults and adolescents [17, 19–22]. Therefore, utilizing peer mentors to deliver health-promoting messages or nutrition education may be an innovative approach to reach underserved and economically disadvantaged populations [22].

Utilizing a peer mentoring model within an EFP and adapted from the concept of a community health educator may be a key strategy in addressing health and wellness in families experiencing food insecurity. The peer mentoring model has been identified as a significant mechanism for helping individuals (both mentor and mentee) develop a sense of purpose and belonging through a supportive relationship, and this also builds and strengthens their social networks [17]. Although peer mentors are recognized for their effectiveness in general community settings, little research has been done to determine the effectiveness of peer-led nutrition interventions in low-income populations using the EFP environment. To address this gap, a nutrition education pilot program was developed using peer mentors in a community EFP setting to teach nutrition education and necessary cooking skills to their fellow community members. Titled *Community Cooks,* this innovative approach of nutrition education in an EFP used a train-the-trainer peer mentor model. This research aimed to identify the successes and challenges of using a peer mentor model within an EFP by conducting focus groups and a qualitative program evaluation. These findings may be used to inform others about the peer mentor model as an approach to deliver nutrition education for EFP community residents.

### Theoretical framework for model development

The authors of this manuscript used two theoretical frameworks to support the program development and implementation: 1) Albert Bandura’s Social Cognitive Theory, and 2) The Socio-Ecological Model. Using Bandura’s Social Cognitive Theory (SCT), the authors theorized that having a peer mentor as a positive role model may increase good nutritional choices and decisions among EFP members [23]. The major component of SCT is delivered through observational learning. SCT encompasses the process of learning desirable behaviors by observing the behavior of others. The observer then replicates these behaviors in order to maximize outcomes. The *Community Cooks* program used peer mentors chosen from the local community to serve as role models for delivering education on healthy food choices, lifestyle, and cooking information to the community at large. This model draws on key components of SCT by then infusing concepts of self-efficacy and through role-modeling by enabling the community members to be able to replicate these behaviors on their own.

The Socio-Ecological Model has also been well established within health and wellness in terms of dietary patterns (e.g., individuals tend to model food choices and behaviors based on social interactions such as children modeling food choices after their parents) thus supporting the model’s use for the *Community Cooks* program [24]. This model notes that individuals, families, and communities influence food choices, and opportunities for health promotion, and disease prevention [25]. Therefore, employing these education strategies within the target audiences’ community will expand not only the reach but also the relevance to community members.

Integrating theoretical frameworks such as SCT and the Socio-Ecological Model within a peer mentoring program is supported when using curricula delivered in regularly scheduled meetings where peer mentors can receive social support, and develop mentoring skills while gaining self-efficacy and influencing behavior change within themselves and others [21, 25, 26].

## Methods

### Setting

Villanova University Fitzpatrick College of Nursing (FCN) and Catholic Social Services (CSS) have a partnership that supported the *Community Cooks* program. CSS’s largest EFP, Martha’s Choice Marketplace (MCM) located in Montgomery County, Pennsylvania [27], serves roughly 900 families each month and provides approximately 800,000 pounds of food annually. As part of the CSS Family Service Center, patrons are provided with a wide array of other empowering services, including, but not limited to, job search assistance, financial literacy classes, nutritional education, and parenting classes [27]. MCM uses a choice market approach which provides families the independence to *shop* the EFP and select from the available foods to promote dignity and reduce stigma. The *Community Cooks* program was conducted from November 2017 through May 2018. Ethics approval was obtained from Villanova University Institutional Review Board, (IRB) and the study was deemed exempt. Written informed consent was obtained from all participants prior to the start of the study. Peer mentors were compensated with monetary cash stipends of 25 dollars per training session attended and 35 dollars for each workshop they assisted in presenting. Peer mentors were not compensated for participation in the focus groups.

### Recruitment

Peer mentors were recruited using flyers, word-of-mouth, and direct referrals from EFP staff who had existing relationships with the MCM volunteers, pantry patrons, or as a member of the Co-Op food share program. Information sessions were also held to recruit potential participants to become mentors. Interested candidates completed a one-page application that included questions about general demographics, availability, and desire to participate in the program. These applications were reviewed by a committee of CSS, MCM, and Villanova University FCN staff to select candidates with demonstrated leadership skills and reliability through their current volunteer roles and affiliations with the EFP. All participants provided written informed consent prior to participation.

### Program design

*Community Cooks* utilized a “train-the-trainer” peer mentor model and involved nine, 60-min, peer mentor training sessions, and three, 60-min, community workshops held at the EFP (Tables 1 and 2). The nine training sessions featured hands-on training and nutrition education provided by the research team with a focus on basic cooking skills, healthy eating principles, and easily prepared recipes that incorporated ingredients commonly available at the EFP. Materials were developed by the research team based on content from the Cooking Matters toolkit, which is tailored towards this population [28]. There were also three peer mentor-led community workshops held at the EFP, allowing the peer mentors to train other pantry patrons in simple, low-cost recipes and basic healthy eating messaging (Tables 1 and 2). The program was set up in a repeating sequence to host three peer mentor training sessions, immediately followed by one live community workshop. This sequence was repeated three times. Three peer mentors rotated as the leaders for the workshops. Responsibilities included facilitating the lesson, demonstrating the recipe preparation, providing samples, and answering community members’ questions. This rotation of peer mentors allowed each mentor an opportunity to teach in front of a live audience. The entire group of peer mentors was present for each community workshop, and the research team was also in attendance to oversee program implementation and ensure content accuracy. Peer mentors also completed a brief multiple-choice quiz of which they needed to achieve an 80% on to ensure proficiency in the content knowledge prior to conducting the workshops.

**Table 1:** Phase I “Community Cooks” Nutrition Education Training Program Curriculum

Lesson	Content	Recipes
Training Session 1	Introduction & Review of Course Content Healthy vs Unhealthy Carbohydrates Portion size matters Choosing wisely-with budget in mind Cook ahead and store to save time Slow cookers-time and money saver	Whole grain recipes
Training Session 2	Budget Stretching using the My Plate Way Review of successes/foods tried Go Lean with Protein /Identifying protein foods Best value proteins/ Plant based proteins	Slow Cooker 3-Bean Chili
Training Session 3	Demonstration & Practice Cooking Session	Whole Grain Recipes Slow Cooker Recipes
Training Session 4	Vary your Vegetables Nutritional benefits Relationship to disease prevention Fresh vs Frozen vs Canned Using herbs and spices to lower sodium	Roasted Winter Squash
Training Session 5	Focus on Fruits Nutritional benefits DASH-benefits of fruit and vegetables: How to select: Seasonal fruits Canned vs Frozen	Fruit recipe
Training Session 6	Demonstration & Practice Cooking Session	Fruit Recipes Vegetable Recipes
Training Session 7	Dairy & Non-Dairy Options Health benefits: calcium, protein, vitamin D Managing lactose intolerance Freezing dairy foods to extend shelf life	Dairy & Non-diary recipe
Training Session 8	Quick and Easy Family Recipes Planning your menu ahead Recipe makeovers Preparing an All-In-One My Plate Meal	A family meal recipe

**Table 2:** Phase I Community Cooks Community Workshop Curriculum

Workshop 1	Part 1: My Plate Can Taste Great! • Identify benefits of whole grains • Identify difference between whole grains and refined grains • Creative use of grains in recipes Part 2. Cooking Demo and Taste Test • Recipe cards for bran flake muffins • Muffin recipe demo preparation and tasting
Workshop 2	Part 1: My Plate Can Taste Great: New Ways to Make Fruit and Veggies • Learn about the health benefits vegetables and fruit provide • How much produce can you buy for $10? • Creative use of fruits and vegetables Part 2. Cooking Demo and Taste Test • Recipe cards for Fruit and Yogurt parfait and Warm Roasted Butternut Squash salad • Fruit and Yogurt Parfait and Butternut Squash salad demo preparation and tasting
Workshop 3	Part 1: My Plate Can Taste Great: Review of All the Food Groups • Learn about the many health benefits all the food groups can provide • Explore ways to save money when buying wholesome, nutritious foods Part 2. Cooking Demo and Taste Test • Recipe cards for Black Bean and Mango Salsa and Butternut Squash Macaroni and Cheese • Greek Yogurt Dip and veggies, and Butternut Square Macaroni and Cheese tasting • Black Bean and Mango Salsa recipe demo preparation and tasting.

### Measures

Two semi-structured focus groups, approximately 60 min in duration, were conducted at the conclusion of the program to elicit feedback from the peer mentors on their perspective of having served as participants in the peer mentor model program. The focus groups were based on guiding questions and had two leaders from the research team assigned to each session (Table 3). All focus group leaders were trained in facilitating focus groups and familiar with conducting qualitative research. The audiotapes were transcribed, qualitative entries were de-identified and then analyzed using descriptive analysis to summarize the findings. Two authors (T.O. and A.M.) independently read and reread the data in order to obtain a broad perspective on the qualitative data as they pertained to each question prompt. Then, the same two authors independently summarized the data to determine key concepts, categories, and themes based on the responses. These authors met again to review and discuss the preliminary findings and to develop final themes. The other authors read the results and indicated agreement with the themes.

**Table 3:** Phase I Community Cooks Focus Group Questions

1. Community Cooks Program-General. Let’s begin by talking generally about Community Cooks and what you thought about the program. a. What did you get out of the program? b. What did you like the best? c. What did you like the least?	
2. Community Cooks Program-Format. Now let’s talk about the Community Cooks program format. a. The format of the Community Cooks Program was to have 3 training sessions conducted followed by 1 workshop conducted by peer mentors. Did you like this format? What worked and what didn’t work for you?	
3. Personal Changes. Now that you have completed the Community Cooks training program, tell us about some of the personal changes that you have made. a. How do you feel about learning to eat healthy and cook healthier now that you’ve gone through the program? b. What are you doing differently with your food choices? c. What are doing differently in food preparation?	
4. Barriers and Challenges. We are aware that there are challenges and barriers to participating in a program like Community Cooks. We need your feedback to identify those challenges and to offer a program that best serves the community. a. We struggled to get people to come to the workshops. Why do you think that happened? b. How do we work around some of the barriers?	
5. Next Steps: Phase 2 Planning. We’re thinking about the next phase of Community Cooks and would like your thoughts as to what we should include.	

## Results

### Socio-demographic characteristics

A total of 31 applications were received and reviewed by the CSS committee and Villanova University FCN research team for potential eligibility. From the total pool of applicants, five individuals were eliminated for not meeting inclusion/exclusion criteria (such as not being available on the selected evening or unable to secure childcare to attend), a 15 individuals were invited to participate, 13 began the program, and 11 participants completed the program. One subject withdrew due to personal health issues, and a second subject withdrew due to transportation issues. After the *Community Cooks* peer mentor training program, the retention rate was 85%. All the peer mentors were women over 40 years-of-age, most were high school graduates and currently participate in some form of federal nutrition assistance programming and appeared to be a representative sample when compared to the total MCM client demographics (Table 4). The 11 subjects who completed the study attended an average of 92.7% collectively of the nine scheduled training sessions (4 participants attended 80%, and 7 participants attended 100% of the sessions).

**Table 4:** Socio-demographic characteristics of peer mentor participants (*n* = 10; 1 missing) compared to sample MCM clients (*n* = 329)

**Characteristic**	**Number (Peer Mentors)**	**Number (MCM Clients)**
**Age (range in years)**		
2–39	0	90 (27%)
40–49	4 (40%)	69 (21%)
50–59	0	92 (28%)
60 and over	6 (60%)	78 (24%)
**Education Level**		
Less than a high-school degree	1 (10%)	28 (9%)
High school degree or GED	3 (30%)	157 (48%)
Technical or vocational school or trade certificate	0	41 (12%)
Some college, but have not graduated	3 (30%)	0
Two-year or Four-year college degree	3 (30%)	83 (25%)
Unknown	0	20 (6%)
**Federal Food Assistance Program Participation (check all that apply)**		
Food Pantry	7 (70%)	329 (100%)
Free or reduced school meals	2 (20%)	Not collected
Summer Feeding Programs	0	33 (10%)
Head Start	2 (20%)	Not collected
Medicaid	6 (60%)	Not collected
SNAP	4 (40%)	165 (50%)
WIC	2 (20%)	Not collected
None reported	2 (20%)	Not collected
**Race/Ethnicity**		
American Indian or Alaska Native	1 (1%)	6 (2%)
Asian	0	1 (< 1%)
Black/African American	4 (40%)	134 (41%)
Hispanic	0	44 (13%)
White	4 (40%)	125 (38%)
Other	1 (1%)	15 (5%)
Unknown	0	4 (1%)

### Peer Mentor feedback

Focus group sessions provided peer mentor input from their perspective as having served as a peer mentor in the *Community Cooks* program, and their feedback was overwhelmingly positive. All the peer mentors (*n* = 11) participated in the focus groups and provided feedback on how the program not only benefited them personally but also on the impact they felt they had on community members. This feedback was valuable in helping researchers understand the appropriateness of the program training, peer mentor satisfaction, and their commitment to the model.

Key successes of the program included: 1) serving in the role as a peer mentor was an empowering experience which gave them a sense of community, purpose, and camaraderie; 2) the nutrition education was appropriately tailored those living with food insecurity; 3) the recipes required minimal cooking skills and included low-cost easily accessible foods available at the EFP. Key challenges of the program were the lack of community member engagement in the nutrition education workshops.

### Empowering peer mentor experience

Peer mentors were asked about their experience in their role serving as a program peer mentor for the *Community Cooks* program. Aspects regarding the peer mentor model, including the train-the-trainer model, with nine training sessions and three community workshops, were explored. The consensus from the peer mentors was that they liked the way the *Community Cooks* program format was designed; they felt the training was delivered in a format that was easy to understand and replicate. They also included that through their training, they felt prepared to conduct each workshop and felt well supported by research staff as well as one another. Feedback included:

“*I think [the format] worked because it gave you more of a backbone to stand on. This way you had enough [information] under your belt that you felt confident enough to do the training instead of just one workshop, one training. You know, you had enough confidence to say okay, I know little bit about this*.”

*“I think [the peer mentor model] boosted confidence level a little bit when you're going to demonstrate, [and] when you're meeting people. It helped you to communicate better [and] I think because it's not just me, it’s me and [Name].”*

*“I would say that I liked how we demonstrate to [other] people.”*

The group members spoke about how they felt more confident in their role as a peer mentor as time progressed and they were becoming more comfortable in their role. Peer mentors shared:

*“I think that the first [workshop] we had [was] okay. But I think each one got better. I really do because everybody felt more confident than seeing the first people up there.”*

Though the purpose of the *Community Cooks* program was to provide peer mentor-led nutrition education and skills to community members, the program also positively influenced many of the peer mentors’ personal health habits. Another critical and noteworthy focus group finding was not just the benefit of the peer mentor model for the EFP and MCM community, but the peer mentors also discussed the positive dynamic and synergy created among the group of peer mentors. They developed a shared level of respect for each other and expressed how, during the training sessions, they learned from one another and were able to share this information later when they were conducting the workshops. They shared:

*“I think the group that we ended up with was a good group because we all had something to put in. We had diversity to put in from other cultures. We had age brackets. We had social brackets.”*

“*We fell right in with each other.”*

*“I can do this.”*

*“Everybody was on the maturity level and the learning level that it made it so that when somebody said something everybody's ears perked up.”*

*“The group that committed themselves to [the program], we learned from each. We were able to feed off each other and broaden it.”*

Peer mentors discussed how they developed a sense of belonging within the group and how they genuinely looked forward to coming to sessions and developing friendships among other peers. They described a cohesive sense of community and camaraderie that were beyond program expectations. This web of community created a bond among them that was unexpected to the peers themselves and to program facilitators. The peer mentors recognized that without the *Community Cooks* program, they would not have met nor created this unique connection among one another. These findings reflect the tangible and intangible power of a peer mentoring model.

### Appropriately tailored nutrition education

The next theme that emerged from the focus group findings was on the reaction the peer mentors had on the content, quality, and appropriateness of the nutrition education program to suit the needs of their community members. Peer mentors indicated that not only did they learn new information from the research team, but that they greatly valued the ideas that were also shared by their fellow peers. Peer mentor comments included:

“*[The program] made it easy to want to eat healthy and learn more. I felt very at home*.”

“*It was very informative, and I got a lot from the class [es]. I'm quite sure others learned a lot too*.”

“*I thought the classes gave a lot of good information. I was able to share with my family, with neighbors.*”

“*This program reinforced a lot of things that I knew, kind of brought back some things that I knew, and it helped me share with others. Like taking something and making it healthier and then having somebody say hey, this is really pretty good.*”

Peer mentors felt motivated to adopt many of the nutrition education strategies for themselves and their family members. The majority of peer mentors shared the personal changes they made in as a result of the program, and responses included:

*“It helped me as a whole think twice before eating things that are full of salt or margarine or you can eat good without frying everything.”*

*“I use less salt and I use more different seasonings.”*

*“I use more like natural herbs. I've used fresh cilantro. I've used fresh garlic where before I was using the canned or the jarred. I got a chopper, and I put the garlic in it and chop the garlic up with a little bit of olive oil and cook with that. I use more olive oil. I use more olive oil than other kind of oils. I try to sweeten with honey instead of sugar.”*

### Recipes included the use of Assessible ingredients with simple preparation

The peer mentors noted an increase in their cooking self-efficacy due to time spent in the training sessions with the research team. They noted that training sessions expanded their knowledge as well as their desire to experiment and try simple new recipes with easily accessible ingredients. Two peer mentors shared their new-found ease with recipe experimentation:

*“It was just like instead of a, b, and c, okay, I can add this to it, or I can add that to it and see how far I could push that envelope”*

*“I was getting a little bit turned off by meat, so this all has taught me to experiment. I was on [to] black beans, and I was introducing them to other family members and telling them about the vitamins. I think I'm being more conscious of my plate with the veggies. I am being creative. I am making my own dressing and trying different things and really introducing the veggies to the grandkids and we're trying a couple of things”.*

### Challenges of peer Mentor model

It was noted throughout the *Community Cooks* program that is was still challenging to attract community members to attend the workshops. Workshop participation averaged approximately five patrons per session. The focus group elicited peer mentors’ reactions regarding the poor attendance by the MCM community as well as any barriers that might have prevented community member attendance. Insights included:

*“I think what the barriers are, it's a foodbank night. That's why. People have a hard time getting here anyway because they're bussing it, cabbing it, Uber-ing it, whatever. So, they have a hard time getting here anyway. Now, to get them here twice a month, oh, you done asked a lot. So, the barrier is the transportation here. The second barrier is they have their food with them. They don’t want to miss out of that food because they need it.”*

*“You’re not going to get everybody, but here are going to be some people who are enticed by “oh, you did this and oh, there’s going to be a drawing and oh, we get to take food, and we cook” Some people may be interested.”*

The peer mentors also shared ideas for future marketing to entice community members to attend, such as raffle drawings and incentives offered for workshop participation. There was consensus around the need to educate the MCM community on the benefits that workshop participation could bestow upon attendees. The peer mentors were also asked about the use of the word “workshop” and whether that word resonated with the community. Peer mentors suggested that this terminology was not ideal and contributed to the lack of community participation. One peer mentor shared: *“I think like cooking demo or something. Do away with the word ‘workshop’. A lot of people have different ideas of [what a workshop is].”* Another peer mentor reinforced this by saying community members may feel: “*[They] might feel they’re getting lectured. Because usually a workshop, a lot of times you get lectured.* There was also uncertainty about the “who, what, when, why” of the workshop, which ultimately led to confusion and limited participation. Overall, the peer mentors felt that calling it ‘events’, ‘demonstrations’, or ‘cooking classes’ over the terminology “workshop” would be preferred.

The peer mentors also envisioned an opportunity to provide nutrition education beyond the scope of a formal workshop and directly to patrons while were waiting in the long lines to shop the EFP. Peer mentors felt that capturing patrons during their shopping trip might be an ideal window of opportunity to share valuable nutrition knowledge before they enter the EFP.

## Discussion

The on-going challenge of food insecurity faced by many American communities highlights the need for more evidence-based nutrition education programs delivered by peer mentors in an EFP [1]. These types of programs can provide a mechanism for reaching individuals with simple healthy eating messages and skills for cooking on a budget that otherwise may not be available to these families. Using a peer mentor-led model to deliver this type of nutrition education to the broader community may be a feasible option and one that was well-received based on peer mentor feedback. Also, based on peer mentor feedback, this education program provided relevant and empowering information to patrons and to the peer mentors themselves, to take charge of their health and food choices in realistic ways, and mindful of food choices and budget. The peer mentor model has also demonstrated success in other areas of health-related education with behavior change and increased levels of knowledge and maybe a key component to engage harder to reach community members [17, 19].

Feedback from our peer mentors supported the peer mentor model, and the *Community Cooks* program implementation. Through the focus groups, the peer mentors expressed that the program was timely, educationally appropriate, specific to the population needs, and was structured to empower them with knowledge and skill to deliver relevant health and nutrition messages to fellow community members. The peer mentors taught the workshop participants the concepts and skills necessary in choosing and then preparing healthy foods available in the food pantry. The *Community Cooks* program had a positive effect on the peer mentors’ ability to relate to community members and developed a web of community and a sense of belonging among the peer mentors that created a vested interest in the program. Even though peer mentors were compensated for participating in the training program, many of them indicated that compensation was not necessary to keep them engaged. Each peer mentor was already affiliated with this pantry in some way and was now able to utilize the skills and knowledge they gained through this program for when they return to their original position (pantry volunteers, Co-op members, etc.) where they can continue to educate pantry patrons.

### Academic and clinical partnerships

The *Community Cooks* program benefited from the successful partnership among the FCN research team, the EFP community site, and their host organization CSS. Clinical, academic, and community partnerships that are successful, and committed to its mission and population served can have demonstrable impact. This program was born out of a common goal of all partners – to address the health knowledge gap within a food-insecure population. A cohesive partnership was key in developing the peer mentor model, and educational programming contributed to its success. Partnering with the EFP allowed our team the opportunity to utilize space to host the project, in addition to providing aid in recruitment, assisting in scheduling/or rescheduling educational program sessions as well as administrative support. When creating community-academic partnerships, consideration must be given to partnership experience, commitment to program goals and outcomes, resources, and space, and a defined delegation of roles and responsibilities to ensure successful program implementation. In this peer mentor model, peer mentors had some form of a pre-established relationship with the parent organization, which secured the trust among the mentors and aided in mentor retention.

### Challenges and lessons learned

Challenges and limitations relating to the peer mentor model in the EFP, as well as lessons learned both through implementation and evaluation, must be considered. The program had several limitations as it was a pilot program and was restricted to one EFP. The EFP had space limitations, which impacted not only the time available for peer mentor training sessions but also limiting the number of potential peer mentor participants. Additional limitations of the *Community Cooks* peer mentor program were that while recruitment and retention of peer mentors were successful, it was very challenging to engage the EFP and MCM community. Furthermore, data from community members or workshop attendees was not collected, which may have provided further details on their perceived successes or challenges of attending the *Community Cooks* program. Lastly, a fidelity assessment was not included in this pilot study and will be included in future phases to evaluate the program implementation.

Hosting the live community workshops facilitated by the peer mentors proved to be challenging in achieving the desired attendance and participation outcome. An unexpected limitation that arose was related to scheduling the workshops, which conflicted with the pantry hours, making it a challenge for patrons to attend the workshop and shop during the same timeframe. Workshop participation averaged five patrons per session, and it was learned that conducting the workshops while the food pantry was open may have been an unintended barrier. Community members did not want to “give up” their spot in the EFP line to attend a workshop, nor did they desire to visit the workshop upon exiting the EFP as they had their groceries and perishable items with them. These limitations and challenges will inform future program development as finding ways to connect with the community are crucial for program success.

### Implications for research and practice

The social determinants of health that include zip code, socioeconomic level, and food security are considered part of what predicts the health status of individuals who attend EFPs [1, 29, 30]. It has been well documented that food insecurity is a chronic stressor of health [31]. Food insecure individuals often consume calorie-laden foods with little fruits and vegetables since the cost is an issue, and gaps in knowledge exist on how to incorporate healthy eating from the foods obtained in EFPs [8, 32]. Many EFPs have expanded their services from supplemental food resources to now include services that recognize the connection between hunger and health and the interplay of social and environmental factors that influence food choice [12]. There is a strong need for food and nutrition education in all venues, and EFPs provide a non-traditional setting for education in which innovation and community partners can work together [14, 15, 32, 33]. The program of using peers as leaders in pantries may be one model of connecting food insecurity and health. To sustain these efforts, EFPs must possess a vested administration and staff to identify members from their community motivated to lead and deploy nutrition education efforts throughout the EFP effectively.

## Conclusion

*Community Cooks* was a peer-led nutrition education and healthy cooking program delivered in an EFP setting targeting a community with a high prevalence of food insecurity. The purpose of this program was to develop an innovative approach to nutrition education in an EFP using a train-the-trainer peer mentor model and conducting qualitative program feedback from the peer mentor participants. The focus group revealed many successes and challenges of utilizing peer mentors to deliver a nutrition education program within an EFP, and these results contribute to the body of research on utilizing peer mentors in low-income communities. While further research and testing of this model are needed, this type of educational delivery can be a model for other community health providers and health personnel working in these types of community settings and EFPs. Future programs and research are still needed to explore the health impact of this program model on peer mentors as well as the patrons and community members who are the recipients of peer mentor-led nutrition education within an emergency food pantry setting.

## Data Availability

Not applicable.

## Abbreviations

EFP: Emergency Food Pantry
SCT: Social Cognitive Theory
CSS: Catholic Social Services
MCM: Martha’s Choice Marketplace
FCN: Fitzpatrick College of Nursing
